# (2*RS*,3*SR*,10*SR*,11*RS*)-3,10-Diphen­oxy-18,21-dioxa-5,8-diaza­penta­cyclo­[20.4.0.0^2,5^.0^8,11^.0^12,17^]hexa­cosa-1(26),12,14,16,22,24-hexa­ene-4,9-dione ethyl acetate hemisolvate

**DOI:** 10.1107/S1600536811030200

**Published:** 2011-07-30

**Authors:** J. Suresh, Natarajan Arumugam, Abdulrahman I. Almansour, Usama Karama, P. L. Nilantha Lakshman

**Affiliations:** a Department of Physics, The Madura College, Madurai 625 011, India; b Department of Chemistry, College of Sciences, King Saud University, PO Box 2455, Riyadh 11451, Saudi Arabia; c Department of Food Science and Technology, University of Ruhuna, Mapalana, Kamburupitiya 81100, Sri Lanka

## Abstract

In the title compound, C_34_H_30_N_2_O_6_·0.5C_4_H_8_O_2_, there are two mol­ecules in the asymmetric unit and the structure is stabilized by C—H⋯O inter­actions. The two nonsolvent mol­ecules of the asymmetric unit are linked together by a weak C—H⋯O hydrogen bond. The ethyl acetate mol­ecule is present as a space filler and does not participate in the hydrogen-bonding network.

## Related literature

For background to the pharmaceutical applications of β-lactam anti­biotics, see: Samarendra *et al.* (1994 ▶); Vaccaro & Davis (1998 ▶); Vaccaro *et al.* (1998 ▶); Borthwick *et al.* (1998 ▶).
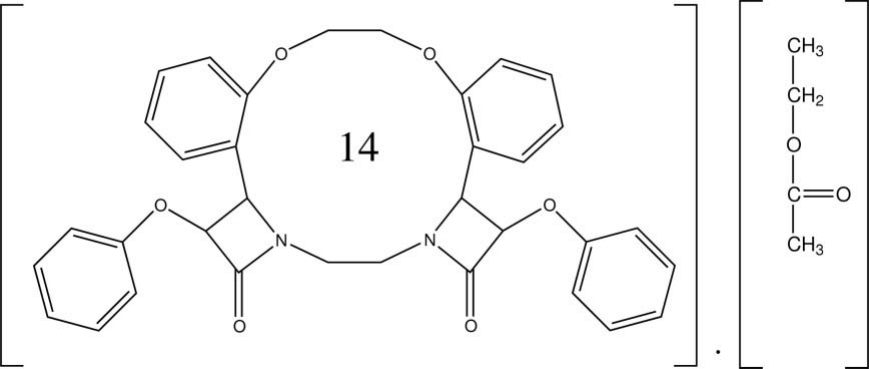


## Experimental

### 

#### Crystal data

C_34_H_30_N_2_O_6_·0.5(C_4_H_8_O_2_)
*M* *_r_* = 606.65
Monoclinic, 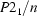

*a* = 18.037 (3) Å
*b* = 17.201 (3) Å
*c* = 21.589 (4) Å
β = 108.722 (1)°
*V* = 6343.67 (19) Å^3^
*Z* = 8
Mo *K*α radiation
μ = 0.09 mm^−1^
*T* = 293 K
0.18 × 0.16 × 0.13 mm

#### Data collection

Bruker SMART APEX CCD diffractometer
Absorption correction: multi-scan (*SADABS*; Bruker, 1998 ▶) *T*_min_ = 0.984, *T*_max_ = 0.988
64493 measured reflections
13208 independent reflections
7192 reflections with *I* > 2σ(*I*)
*R*_int_ = 0.046

#### Refinement

*R*[*F* ^2^ > 2σ(*F* ^2^)] = 0.053
*wR*(*F* ^2^) = 0.175
*S* = 1.02
13208 reflections
813 parameters
H-atom parameters constrained
Δρ_max_ = 0.32 e Å^−3^
Δρ_min_ = −0.24 e Å^−3^

### 

Data collection: *SMART* (Bruker, 2001 ▶); cell refinement: *SAINT* (Bruker, 2001 ▶); data reduction: *SAINT*; program(s) used to solve structure: *SHELXS97* (Sheldrick, 2008 ▶); program(s) used to refine structure: *SHELXL97* (Sheldrick, 2008 ▶); molecular graphics: *PLATON* (Spek, 2009 ▶); software used to prepare material for publication: *SHELXL97*.

## Supplementary Material

Crystal structure: contains datablock(s) global, I. DOI: 10.1107/S1600536811030200/lw2069sup1.cif

Structure factors: contains datablock(s) I. DOI: 10.1107/S1600536811030200/lw2069Isup2.hkl

Supplementary material file. DOI: 10.1107/S1600536811030200/lw2069Isup3.cml

Additional supplementary materials:  crystallographic information; 3D view; checkCIF report

## Figures and Tables

**Table 1:** Hydrogen-bond geometry (Å, °)

*D*—H⋯*A*	*D*—H	H⋯*A*	*D*⋯*A*	*D*—H⋯*A*
C53—H53⋯O6	0.93	2.52	3.344 (3)	147
C2—H2⋯O2	0.93	2.42	3.250 (3)	149
C32—H32⋯O1	0.93	2.33	3.178 (3)	151
C66—H66⋯O7	0.93	2.48	3.299 (3)	147

**Fig. 1.**
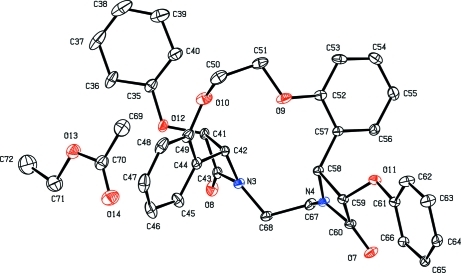
One of the molecule of the two independent molecules and the solvent molecule ethyl acetate of the asymmetric unit(I), showing 10% probability displacement ellipsoids and the atom-numbering scheme. H-atoms are omitted for clarity.

**Fig. 2.**
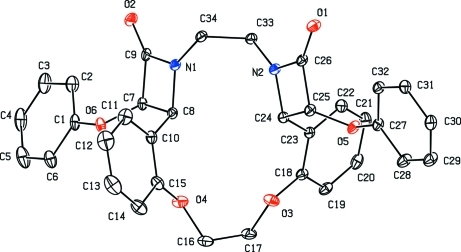
The other molecule of the two independent molecules of the asymmetric unit(I), showing 10% probability displacement ellipsoids and the atom-numbering scheme. H-atoms are omitted for clarity.

## supplementary crystallographic information

### Comment

β-Lactam antibiotics are widely employed in the treatment of bacterial infections(Samarendra *et al.*, 1994). In recent years, several natural β-lactams have been shown to exhibit a high antibacterial activity. 1,3,4-trisubstituted β-lactams were found to be new, potent cholesterol absorption inhibitors (Vaccaro & Davis 1998; Vaccaro *et al.*, 1998) human cytomegalovirus (Borthwick *et al.*, 1998). Furthermore, many anticancer drugs in current use are toxic and thus limited in their efficacy. It is therefore essential to develop novel chemotherapeutic agents with lower levels of toxicity. β-lactam antibiotics have been used for many years with limited or no toxicity. In view of this, the crystal structure determination of the title compound was carried out.

The asymmetric unit of (I) contain two independent molecules, The pair have almost identical geometry. The internal angles of the four-membered rings of the title compound, (I), vary from 85.3 (8) to 96.2 (5)° . The sums of the bond angles at the N atoms are 359.4 (2) and 360.0 (1),359.3 (8) 359.9 (5) for N1, N2,N3 and N4 respectively are in accordance with *sp*^2^ hybridization. The conformation of the molecules is stabilized by weak intramolecular C—H···O and C—H···N interactions, (Table 1). The two non-solvent molecules of the asymmetruc unit are linked together by a weak C—H···O hydrogen bond. The presence of the ethyl acetate solvent as a mere space filler suggests that its contribution to the intermolecular interactions is insignificant but it has nevertheless played a role in crystallization. There is a solvent accessible void of 66.7 Å^3^ in the structure.

### Experimental

A solution of phenoxyacetyl chloride in dry dichloromethane (20 ml) was slowly added to a solution of bisimine (1 mmol) and Et_3_N (3.5 mmol) in dichloromethane (20 ml) at 0 °C and the reaction mixture was stirred for 12 h at room temperature. Completion of the reaction evidenced by TLC analysis, the reaction mixture was washed with water (2 *X* 20 ml), saturated NaHCO_3_ (15 ml) and brine (15 ml). The organic layer was dried over anhydrous Na_2_SO_4_ and concentrated under reduced pressure. The crude product was separated by column chromatography (hexane: ethyl acetate 7:3) gave pure bis-β-lactam in good yield. The product was recrystallized from ethyl acetate by slow evaporation technique. m.p.: 195°C, yield: 48%

### Refinement

The H atoms were placed in calculated positions and allowed to ride on their carrier atoms with C—H = 0.93–0.98 Å and *U*_iso_ = 1.2*U*_eq_(C,) for CH, CH_2_ groups and *U*_iso_ = 1.5*U*_eq_(C) for CH_3_ group.

### Crystal data

**Table:**

C_34_H_30_N_2_O_6_·0.5(C_4_H_8_O_2_)	*F*(000) = 2560
*M**_r_* = 606.65	*D*_x_ = 1.270 Mg m^−^^3^
Monoclinic, *P*2_1_/*n*	Mo *K*α radiation, λ = 0.71075 Å
Hall symbol: -P 2yn	Cell parameters from 2500 reflections
*a* = 18.037 (3) Å	θ = 2–27°
*b* = 17.201 (3) Å	µ = 0.09 mm^−^^1^
*c* = 21.589 (4) Å	*T* = 293 K
β = 108.722 (10)°	Block, colourless
*V* = 6343.67 (19) Å^3^	0.18 × 0.16 × 0.13 mm
*Z* = 8	

### Data collection

**Table:**

Bruker SMART APEX CCD diffractometer	13208 independent reflections
Radiation source: fine-focus sealed tube	7192 reflections with *I* > 2σ(*I*)
graphite	*R*_int_ = 0.046
ω scans	θ_max_ = 26.6°, θ_min_ = 1.7°
Absorption correction: multi-scan (*SADABS*; Bruker, 1998)	*h* = −22→22
*T*_min_ = 0.984, *T*_max_ = 0.988	*k* = −21→21
64493 measured reflections	*l* = −27→27

### Refinement

**Table:**

Refinement on *F*^2^	Primary atom site location: structure-invariant direct methods
Least-squares matrix: full	Secondary atom site location: difference Fourier map
*R*[*F*^2^ > 2σ(*F*^2^)] = 0.053	Hydrogen site location: inferred from neighbouring sites
*wR*(*F*^2^) = 0.175	H-atom parameters constrained
*S* = 1.02	*w* = 1/[σ^2^(*F*_o_^2^) + (0.0801*P*)^2^ + 1.3813*P*] where *P* = (*F*_o_^2^ + 2*F*_c_^2^)/3
13208 reflections	(Δ/σ)_max_ < 0.001
813 parameters	Δρ_max_ = 0.32 e Å^−^^3^
0 restraints	Δρ_min_ = −0.24 e Å^−^^3^

### Special details

**Table:**

Geometry. All e.s.d.'s (except the e.s.d. in the dihedral angle between two l.s. planes) are estimated using the full covariance matrix. The cell e.s.d.'s are taken into account individually in the estimation of e.s.d.'s in distances, angles and torsion angles; correlations between e.s.d.'s in cell parameters are only used when they are defined by crystal symmetry. An approximate (isotropic) treatment of cell e.s.d.'s is used for estimating e.s.d.'s involving l.s. planes.
Refinement. Refinement of *F*^2^ against ALL reflections. The weighted *R*-factor *wR* and goodness of fit *S* are based on *F*^2^, conventional *R*-factors *R* are based on *F*, with *F* set to zero for negative *F*^2^. The threshold expression of *F*^2^ > σ(*F*^2^) is used only for calculating *R*-factors(gt) *etc*. and is not relevant to the choice of reflections for refinement. *R*-factors based on *F*^2^ are statistically about twice as large as those based on *F*, and *R*- factors based on ALL data will be even larger.

### Fractional atomic coordinates and isotropic or equivalent isotropic displacement parameters (Å^2^)

**Table:**

	*x*	*y*	*z*	*U*_iso_*/*U*_eq_	
O1	0.58820 (10)	0.50849 (11)	0.49402 (8)	0.0675 (5)	
O2	0.46252 (10)	0.40807 (11)	0.76094 (9)	0.0678 (5)	
O3	0.79089 (11)	0.27095 (10)	0.67869 (9)	0.0692 (5)	
O4	0.68980 (10)	0.18393 (11)	0.72407 (9)	0.0723 (5)	
O5	0.75451 (8)	0.41331 (10)	0.52020 (7)	0.0543 (4)	
O6	0.54253 (9)	0.23356 (9)	0.79798 (7)	0.0546 (4)	
N1	0.53463 (10)	0.36778 (11)	0.69396 (9)	0.0482 (5)	
N2	0.62448 (10)	0.43556 (11)	0.59046 (8)	0.0477 (5)	
C1	0.53443 (14)	0.23039 (16)	0.85949 (11)	0.0579 (7)	
C2	0.50667 (17)	0.29204 (19)	0.88634 (13)	0.0738 (8)	
H2	0.4938	0.3387	0.8636	0.089*	
C3	0.49799 (19)	0.2840 (2)	0.94753 (15)	0.0934 (11)	
H3	0.4790	0.3253	0.9658	0.112*	
C4	0.5172 (2)	0.2159 (3)	0.98101 (16)	0.1071 (14)	
H4	0.5124	0.2110	1.0225	0.129*	
C5	0.5436 (2)	0.1545 (3)	0.95333 (17)	0.1107 (13)	
H5	0.5555	0.1078	0.9760	0.133*	
C6	0.55309 (18)	0.16067 (19)	0.89231 (14)	0.0805 (9)	
H6	0.5715	0.1190	0.8740	0.097*	
C7	0.56993 (12)	0.30462 (13)	0.78045 (10)	0.0451 (5)	
H7	0.6143	0.3252	0.8160	0.054*	
C8	0.58797 (12)	0.30110 (13)	0.71428 (10)	0.0438 (5)	
H8	0.6423	0.3153	0.7197	0.053*	
C9	0.51115 (12)	0.36898 (14)	0.74746 (11)	0.0481 (6)	
C10	0.56317 (14)	0.22914 (14)	0.67387 (10)	0.0501 (6)	
C11	0.48777 (17)	0.2166 (2)	0.63373 (13)	0.0766 (8)	
H11	0.4499	0.2545	0.6302	0.092*	
C12	0.4673 (2)	0.1484 (3)	0.59849 (16)	0.1033 (12)	
H12	0.4158	0.1405	0.5721	0.124*	
C13	0.5221 (3)	0.0930 (2)	0.60227 (17)	0.1031 (12)	
H13	0.5079	0.0477	0.5778	0.124*	
C14	0.5990 (2)	0.10279 (18)	0.64218 (15)	0.0818 (9)	
H14	0.6365	0.0649	0.6444	0.098*	
C15	0.61848 (16)	0.17077 (15)	0.67866 (12)	0.0593 (6)	
C16	0.75889 (17)	0.15158 (17)	0.71566 (17)	0.0795 (9)	
H16A	0.7482	0.1332	0.6712	0.095*	
H16B	0.7776	0.1082	0.7453	0.095*	
C17	0.81814 (16)	0.21491 (17)	0.73031 (15)	0.0751 (8)	
H17A	0.8231	0.2385	0.7722	0.090*	
H17B	0.8688	0.1946	0.7317	0.090*	
C18	0.81363 (13)	0.34669 (15)	0.69163 (10)	0.0488 (6)	
C19	0.88309 (14)	0.36788 (17)	0.73889 (11)	0.0582 (7)	
H19	0.9157	0.3301	0.7645	0.070*	
C20	0.90333 (15)	0.4447 (2)	0.74750 (13)	0.0669 (8)	
H20	0.9498	0.4589	0.7793	0.080*	
C21	0.85609 (17)	0.50128 (18)	0.70996 (13)	0.0675 (7)	
H21	0.8704	0.5534	0.7161	0.081*	
C22	0.78640 (15)	0.47960 (15)	0.66253 (11)	0.0559 (6)	
H22	0.7543	0.5177	0.6370	0.067*	
C23	0.76414 (12)	0.40245 (13)	0.65274 (10)	0.0426 (5)	
C24	0.68875 (12)	0.37855 (13)	0.60175 (10)	0.0439 (5)	
H24	0.6725	0.3259	0.6090	0.053*	
C25	0.68248 (12)	0.39336 (14)	0.52855 (10)	0.0466 (5)	
H25	0.6570	0.3500	0.5002	0.056*	
C26	0.62359 (12)	0.45784 (15)	0.53028 (10)	0.0482 (6)	
C27	0.75411 (12)	0.42248 (14)	0.45610 (10)	0.0476 (5)	
C28	0.81786 (15)	0.39355 (18)	0.44128 (12)	0.0685 (8)	
H28	0.8577	0.3679	0.4730	0.082*	
C29	0.82196 (16)	0.40302 (18)	0.37899 (13)	0.0721 (8)	
H29	0.8649	0.3837	0.3690	0.087*	
C30	0.76339 (15)	0.44063 (16)	0.33156 (12)	0.0611 (7)	
H30	0.7662	0.4462	0.2895	0.073*	
C31	0.70102 (14)	0.46967 (15)	0.34697 (11)	0.0546 (6)	
H31	0.6615	0.4957	0.3152	0.066*	
C32	0.69594 (13)	0.46082 (14)	0.40911 (11)	0.0507 (6)	
H32	0.6532	0.4808	0.4190	0.061*	
C33	0.58264 (13)	0.46822 (14)	0.63199 (11)	0.0504 (6)	
H33A	0.5624	0.5188	0.6149	0.061*	
H33B	0.6195	0.4760	0.6755	0.061*	
C34	0.51501 (12)	0.41862 (15)	0.63752 (11)	0.0526 (6)	
H34A	0.4729	0.4528	0.6391	0.063*	
H34B	0.4956	0.3871	0.5983	0.063*	
O7	0.08612 (10)	−0.03084 (12)	0.50260 (9)	0.0732 (6)	
O8	−0.02514 (11)	0.08467 (11)	0.77821 (10)	0.0750 (5)	
O9	0.27029 (12)	0.22602 (11)	0.67684 (9)	0.0814 (6)	
O10	0.18251 (14)	0.31822 (12)	0.73090 (10)	0.0873 (6)	
O11	0.23893 (8)	0.08131 (10)	0.51517 (7)	0.0545 (4)	
O12	0.05351 (10)	0.25116 (10)	0.81692 (8)	0.0634 (5)	
N3	0.03988 (10)	0.12389 (12)	0.70535 (9)	0.0516 (5)	
N4	0.11730 (10)	0.05203 (11)	0.59366 (8)	0.0495 (5)	
C35	0.10712 (19)	0.29037 (16)	0.86937 (13)	0.0687 (7)	
C36	0.0732 (3)	0.33826 (19)	0.90397 (16)	0.1016 (12)	
H36	0.0191	0.3433	0.8925	0.122*	
C37	0.1232 (4)	0.3788 (3)	0.9568 (2)	0.146 (2)	
H37	0.1017	0.4126	0.9801	0.176*	
C38	0.2030 (4)	0.3706 (3)	0.9758 (3)	0.158 (2)	
H38	0.2352	0.3967	1.0123	0.190*	
C39	0.2332 (3)	0.3238 (3)	0.9400 (2)	0.1273 (15)	
H39	0.2873	0.3184	0.9520	0.153*	
C40	0.1864 (2)	0.2834 (2)	0.88599 (16)	0.0918 (10)	
H40	0.2087	0.2522	0.8616	0.110*	
C41	0.08090 (14)	0.18611 (14)	0.79130 (11)	0.0506 (6)	
H41	0.1279	0.1642	0.8232	0.061*	
C42	0.09050 (13)	0.19243 (13)	0.72190 (11)	0.0471 (5)	
H42	0.1441	0.1811	0.7228	0.057*	
C43	0.02096 (14)	0.12327 (14)	0.76097 (11)	0.0521 (6)	
C44	0.05858 (15)	0.26504 (16)	0.68418 (11)	0.0562 (6)	
C45	−0.01947 (17)	0.2737 (2)	0.64774 (13)	0.0765 (9)	
H45	−0.0540	0.2326	0.6448	0.092*	
C46	−0.0471 (2)	0.3434 (3)	0.61535 (15)	0.1036 (14)	
H46	−0.1000	0.3491	0.5921	0.124*	
C47	0.0032 (3)	0.4026 (3)	0.61783 (17)	0.1100 (15)	
H47	−0.0149	0.4479	0.5944	0.132*	
C48	0.0809 (3)	0.39636 (19)	0.65468 (16)	0.1004 (12)	
H48	0.1149	0.4377	0.6571	0.120*	
C49	0.1083 (2)	0.32766 (17)	0.68836 (13)	0.0713 (8)	
C50	0.2471 (2)	0.34862 (19)	0.71346 (19)	0.0998 (11)	
H50A	0.2698	0.3930	0.7406	0.120*	
H50B	0.2299	0.3650	0.6681	0.120*	
C51	0.3058 (2)	0.2846 (2)	0.72398 (17)	0.0935 (10)	
H51A	0.3535	0.3034	0.7177	0.112*	
H51B	0.3184	0.2640	0.7680	0.112*	
C52	0.30210 (13)	0.15310 (15)	0.68510 (11)	0.0536 (6)	
C53	0.37626 (15)	0.1362 (2)	0.72771 (13)	0.0705 (8)	
H53	0.4073	0.1757	0.7522	0.085*	
C54	0.40356 (16)	0.0618 (2)	0.73372 (14)	0.0803 (9)	
H54	0.4531	0.0508	0.7625	0.096*	
C55	0.35892 (18)	0.0031 (2)	0.69790 (13)	0.0774 (9)	
H55	0.3780	−0.0476	0.7020	0.093*	
C56	0.28482 (15)	0.01966 (16)	0.65534 (11)	0.0592 (6)	
H56	0.2542	−0.0204	0.6313	0.071*	
C57	0.25554 (12)	0.09451 (13)	0.64801 (10)	0.0427 (5)	
C58	0.17667 (12)	0.11307 (13)	0.59957 (10)	0.0436 (5)	
H58	0.1580	0.1652	0.6055	0.052*	
C59	0.16737 (12)	0.09423 (14)	0.52655 (10)	0.0474 (5)	
H59	0.1360	0.1338	0.4969	0.057*	
C60	0.11648 (13)	0.02564 (16)	0.53458 (11)	0.0522 (6)	
C61	0.23611 (12)	0.07508 (14)	0.45046 (10)	0.0461 (5)	
C62	0.29714 (16)	0.10742 (19)	0.43439 (13)	0.0752 (9)	
H62	0.3367	0.1339	0.4658	0.090*	
C63	0.29951 (17)	0.10048 (19)	0.37165 (13)	0.0788 (9)	
H63	0.3409	0.1225	0.3609	0.095*	
C64	0.24212 (15)	0.06174 (15)	0.32478 (11)	0.0571 (6)	
H64	0.2443	0.0574	0.2825	0.069*	
C65	0.18145 (14)	0.02940 (14)	0.34073 (11)	0.0516 (6)	
H65	0.1422	0.0028	0.3092	0.062*	
C66	0.17816 (13)	0.03615 (14)	0.40402 (10)	0.0489 (6)	
H66	0.1367	0.0143	0.4147	0.059*	
C67	0.08087 (14)	0.02224 (15)	0.63947 (12)	0.0567 (6)	
H67A	0.1210	0.0153	0.6815	0.068*	
H67B	0.0591	−0.0286	0.6246	0.068*	
C68	0.01636 (13)	0.07316 (17)	0.64897 (12)	0.0628 (7)	
H68A	−0.0050	0.1049	0.6101	0.075*	
H68B	−0.0253	0.0397	0.6526	0.075*	
O13	0.64589 (13)	0.69087 (14)	0.51650 (12)	0.1002 (7)	
O14	0.67151 (18)	0.64668 (18)	0.61737 (13)	0.1269 (9)	
C69	0.5398 (2)	0.6845 (2)	0.5557 (2)	0.1183 (13)	
H69A	0.5112	0.6431	0.5285	0.178*	
H69B	0.5238	0.7333	0.5340	0.178*	
H69C	0.5293	0.6843	0.5965	0.178*	
C70	0.6249 (2)	0.6732 (2)	0.56792 (18)	0.0894 (10)	
C71	0.7275 (2)	0.6741 (3)	0.5224 (2)	0.1394 (17)	
H71A	0.7626	0.7059	0.5564	0.167*	
H71B	0.7394	0.6198	0.5333	0.167*	
C72	0.7356 (3)	0.6917 (4)	0.4603 (3)	0.187 (3)	
H72A	0.6979	0.6625	0.4268	0.280*	
H72B	0.7874	0.6782	0.4607	0.280*	
H72C	0.7270	0.7463	0.4515	0.280*	

### Atomic displacement parameters (Å^2^)

**Table:**

	*U*^11^	*U*^22^	*U*^33^	*U*^12^	*U*^13^	*U*^23^
O1	0.0629 (10)	0.0937 (14)	0.0532 (10)	0.0301 (10)	0.0288 (8)	0.0327 (10)
O2	0.0679 (11)	0.0809 (13)	0.0716 (12)	0.0114 (10)	0.0459 (10)	0.0080 (10)
O3	0.0805 (12)	0.0558 (12)	0.0588 (11)	0.0143 (10)	0.0050 (9)	0.0102 (9)
O4	0.0673 (11)	0.0669 (12)	0.0752 (13)	0.0155 (9)	0.0121 (10)	−0.0115 (10)
O5	0.0451 (8)	0.0895 (12)	0.0327 (8)	0.0076 (8)	0.0188 (7)	0.0068 (8)
O6	0.0673 (10)	0.0610 (10)	0.0375 (8)	−0.0207 (8)	0.0196 (7)	0.0042 (7)
N1	0.0469 (10)	0.0603 (12)	0.0444 (11)	0.0092 (9)	0.0243 (8)	0.0148 (9)
N2	0.0472 (10)	0.0637 (12)	0.0382 (10)	0.0142 (9)	0.0219 (8)	0.0149 (9)
C1	0.0575 (14)	0.0792 (19)	0.0360 (13)	−0.0279 (13)	0.0135 (11)	0.0048 (13)
C2	0.0846 (19)	0.093 (2)	0.0494 (16)	−0.0148 (17)	0.0288 (14)	0.0006 (15)
C3	0.103 (2)	0.133 (3)	0.0557 (19)	−0.039 (2)	0.0420 (17)	−0.010 (2)
C4	0.121 (3)	0.161 (4)	0.0485 (19)	−0.064 (3)	0.0388 (19)	0.002 (2)
C5	0.145 (3)	0.122 (3)	0.065 (2)	−0.044 (3)	0.033 (2)	0.030 (2)
C6	0.099 (2)	0.083 (2)	0.0595 (18)	−0.0216 (18)	0.0262 (16)	0.0180 (16)
C7	0.0476 (12)	0.0528 (14)	0.0373 (12)	−0.0109 (11)	0.0171 (9)	0.0042 (10)
C8	0.0429 (11)	0.0527 (14)	0.0388 (12)	0.0010 (10)	0.0173 (9)	0.0097 (10)
C9	0.0450 (12)	0.0607 (15)	0.0455 (13)	−0.0054 (11)	0.0242 (10)	0.0057 (11)
C10	0.0571 (14)	0.0587 (15)	0.0357 (12)	−0.0058 (12)	0.0168 (10)	0.0061 (11)
C11	0.0685 (17)	0.097 (2)	0.0555 (17)	−0.0120 (16)	0.0076 (14)	−0.0075 (16)
C12	0.102 (3)	0.122 (3)	0.065 (2)	−0.032 (3)	−0.0015 (18)	−0.015 (2)
C13	0.146 (4)	0.083 (3)	0.068 (2)	−0.034 (3)	0.016 (2)	−0.0200 (19)
C14	0.118 (3)	0.0595 (19)	0.0642 (19)	−0.0030 (18)	0.0242 (18)	−0.0039 (15)
C15	0.0758 (17)	0.0550 (16)	0.0455 (14)	−0.0040 (14)	0.0169 (13)	0.0033 (12)
C16	0.083 (2)	0.0571 (18)	0.100 (2)	0.0284 (16)	0.0317 (17)	0.0122 (16)
C17	0.0671 (17)	0.0720 (19)	0.081 (2)	0.0288 (16)	0.0170 (15)	0.0269 (16)
C18	0.0508 (13)	0.0615 (16)	0.0386 (12)	0.0093 (12)	0.0209 (10)	0.0029 (11)
C19	0.0480 (13)	0.085 (2)	0.0441 (14)	0.0124 (13)	0.0180 (11)	0.0007 (13)
C20	0.0535 (15)	0.104 (2)	0.0479 (15)	−0.0090 (16)	0.0226 (12)	−0.0068 (15)
C21	0.0807 (19)	0.0758 (19)	0.0549 (16)	−0.0181 (16)	0.0343 (15)	−0.0066 (15)
C22	0.0660 (15)	0.0625 (17)	0.0456 (14)	0.0029 (13)	0.0268 (12)	0.0063 (12)
C23	0.0492 (12)	0.0527 (14)	0.0335 (11)	0.0052 (11)	0.0237 (10)	0.0066 (10)
C24	0.0487 (12)	0.0501 (13)	0.0368 (12)	0.0055 (10)	0.0193 (10)	0.0067 (10)
C25	0.0458 (12)	0.0620 (15)	0.0340 (11)	0.0037 (11)	0.0157 (9)	0.0049 (10)
C26	0.0426 (12)	0.0682 (16)	0.0375 (12)	0.0073 (11)	0.0182 (10)	0.0117 (11)
C27	0.0485 (12)	0.0650 (15)	0.0334 (12)	−0.0013 (11)	0.0191 (10)	0.0009 (11)
C28	0.0596 (15)	0.105 (2)	0.0479 (15)	0.0214 (15)	0.0276 (12)	0.0132 (14)
C29	0.0701 (17)	0.102 (2)	0.0581 (17)	0.0154 (16)	0.0402 (14)	0.0052 (15)
C30	0.0754 (17)	0.0769 (18)	0.0411 (14)	−0.0081 (14)	0.0327 (13)	0.0011 (13)
C31	0.0622 (15)	0.0638 (16)	0.0398 (13)	−0.0039 (12)	0.0193 (11)	0.0055 (11)
C32	0.0508 (13)	0.0649 (16)	0.0410 (13)	0.0046 (12)	0.0211 (10)	0.0044 (11)
C33	0.0545 (13)	0.0589 (15)	0.0471 (13)	0.0135 (11)	0.0293 (11)	0.0126 (11)
C34	0.0445 (12)	0.0699 (16)	0.0481 (13)	0.0143 (11)	0.0216 (10)	0.0211 (12)
O7	0.0749 (12)	0.0944 (14)	0.0594 (11)	−0.0356 (11)	0.0341 (9)	−0.0355 (10)
O8	0.0878 (13)	0.0758 (13)	0.0845 (14)	−0.0058 (11)	0.0600 (11)	−0.0011 (10)
O9	0.0924 (14)	0.0577 (13)	0.0716 (13)	−0.0242 (11)	−0.0050 (10)	−0.0041 (10)
O10	0.1087 (16)	0.0700 (13)	0.0707 (14)	−0.0264 (12)	0.0113 (12)	0.0074 (11)
O11	0.0440 (8)	0.0917 (13)	0.0311 (8)	−0.0079 (8)	0.0167 (6)	−0.0012 (8)
O12	0.0800 (11)	0.0635 (11)	0.0474 (10)	0.0159 (9)	0.0216 (9)	−0.0108 (8)
N3	0.0498 (11)	0.0667 (13)	0.0455 (11)	−0.0117 (10)	0.0253 (9)	−0.0141 (9)
N4	0.0491 (10)	0.0665 (13)	0.0386 (10)	−0.0179 (9)	0.0223 (8)	−0.0150 (9)
C35	0.103 (2)	0.0580 (17)	0.0463 (15)	−0.0021 (16)	0.0264 (15)	0.0010 (13)
C36	0.167 (3)	0.077 (2)	0.073 (2)	0.003 (2)	0.057 (2)	−0.0237 (18)
C37	0.249 (6)	0.098 (3)	0.100 (4)	−0.018 (4)	0.066 (4)	−0.048 (3)
C38	0.229 (7)	0.122 (4)	0.094 (4)	−0.050 (5)	0.012 (4)	−0.036 (3)
C39	0.138 (4)	0.112 (3)	0.104 (3)	−0.036 (3)	0.000 (3)	−0.011 (3)
C40	0.104 (3)	0.084 (2)	0.075 (2)	−0.008 (2)	0.0113 (19)	−0.0140 (18)
C41	0.0604 (14)	0.0524 (14)	0.0397 (13)	0.0122 (11)	0.0171 (11)	−0.0046 (11)
C42	0.0459 (12)	0.0529 (14)	0.0460 (13)	0.0001 (11)	0.0196 (10)	−0.0070 (11)
C43	0.0567 (14)	0.0582 (15)	0.0508 (14)	0.0069 (12)	0.0304 (11)	−0.0009 (11)
C44	0.0662 (16)	0.0708 (18)	0.0381 (13)	0.0129 (14)	0.0256 (11)	−0.0018 (12)
C45	0.0727 (18)	0.117 (3)	0.0509 (16)	0.0300 (18)	0.0350 (14)	0.0157 (16)
C46	0.113 (3)	0.161 (4)	0.0489 (18)	0.076 (3)	0.0432 (19)	0.030 (2)
C47	0.184 (5)	0.101 (3)	0.053 (2)	0.066 (3)	0.048 (3)	0.018 (2)
C48	0.183 (4)	0.055 (2)	0.058 (2)	0.014 (2)	0.032 (2)	0.0022 (16)
C49	0.107 (2)	0.0581 (18)	0.0475 (16)	0.0031 (17)	0.0233 (16)	−0.0022 (13)
C50	0.130 (3)	0.061 (2)	0.105 (3)	−0.048 (2)	0.034 (2)	−0.0126 (19)
C51	0.097 (2)	0.073 (2)	0.097 (3)	−0.045 (2)	0.0135 (19)	−0.0191 (19)
C52	0.0526 (14)	0.0641 (17)	0.0454 (14)	−0.0168 (12)	0.0172 (11)	0.0019 (12)
C53	0.0490 (15)	0.107 (3)	0.0533 (16)	−0.0250 (16)	0.0127 (12)	−0.0007 (16)
C54	0.0500 (16)	0.137 (3)	0.0536 (17)	0.0135 (19)	0.0158 (13)	0.0057 (19)
C55	0.084 (2)	0.101 (2)	0.0478 (16)	0.0341 (19)	0.0209 (15)	0.0052 (16)
C56	0.0702 (16)	0.0669 (18)	0.0413 (13)	0.0053 (13)	0.0189 (12)	−0.0057 (12)
C57	0.0461 (12)	0.0563 (15)	0.0304 (11)	−0.0085 (11)	0.0188 (9)	−0.0011 (10)
C58	0.0445 (12)	0.0527 (14)	0.0359 (11)	−0.0063 (10)	0.0163 (9)	−0.0038 (10)
C59	0.0430 (12)	0.0665 (16)	0.0340 (12)	−0.0027 (11)	0.0143 (9)	−0.0018 (11)
C60	0.0435 (12)	0.0775 (18)	0.0381 (12)	−0.0106 (12)	0.0167 (10)	−0.0148 (12)
C61	0.0471 (12)	0.0612 (15)	0.0330 (11)	−0.0030 (11)	0.0169 (10)	0.0027 (10)
C62	0.0686 (17)	0.114 (2)	0.0489 (15)	−0.0386 (17)	0.0273 (13)	−0.0134 (15)
C63	0.0793 (19)	0.116 (3)	0.0559 (17)	−0.0335 (18)	0.0419 (15)	−0.0120 (16)
C64	0.0710 (16)	0.0698 (17)	0.0387 (13)	0.0068 (13)	0.0291 (12)	0.0011 (12)
C65	0.0555 (13)	0.0596 (15)	0.0407 (13)	0.0043 (12)	0.0171 (10)	−0.0047 (11)
C66	0.0466 (12)	0.0619 (15)	0.0415 (13)	−0.0039 (11)	0.0188 (10)	0.0009 (11)
C67	0.0663 (15)	0.0621 (16)	0.0539 (14)	−0.0193 (12)	0.0365 (12)	−0.0153 (12)
C68	0.0493 (13)	0.091 (2)	0.0537 (15)	−0.0201 (13)	0.0249 (11)	−0.0268 (14)
O13	0.0974 (16)	0.1138 (19)	0.0950 (17)	0.0069 (14)	0.0389 (13)	0.0220 (14)
O14	0.144 (2)	0.143 (3)	0.0833 (18)	0.0065 (19)	0.0226 (17)	0.0098 (17)
C69	0.123 (3)	0.116 (3)	0.137 (4)	0.025 (3)	0.071 (3)	0.013 (3)
C70	0.117 (3)	0.080 (2)	0.073 (2)	−0.003 (2)	0.032 (2)	0.0031 (18)
C71	0.096 (3)	0.187 (5)	0.148 (4)	0.013 (3)	0.056 (3)	0.046 (4)
C72	0.139 (4)	0.261 (7)	0.190 (6)	0.011 (4)	0.094 (4)	0.060 (5)

### Geometric parameters (Å, °)

**Table:**

O1—C26	1.208 (3)	O11—C59	1.407 (2)
O2—C9	1.213 (3)	O12—C35	1.403 (3)
O3—C18	1.368 (3)	O12—C41	1.406 (3)
O3—C17	1.436 (3)	N3—C43	1.350 (3)
O4—C15	1.363 (3)	N3—C68	1.446 (3)
O4—C16	1.428 (3)	N3—C42	1.463 (3)
O5—C27	1.390 (2)	N4—C60	1.349 (3)
O5—C25	1.410 (2)	N4—C67	1.446 (3)
O6—C1	1.383 (3)	N4—C58	1.476 (3)
O6—C7	1.415 (2)	C35—C40	1.364 (4)
N1—C9	1.352 (3)	C35—C36	1.380 (4)
N1—C34	1.448 (3)	C36—C37	1.394 (6)
N1—C8	1.471 (3)	C36—H36	0.9300
N2—C26	1.350 (3)	C37—C38	1.370 (7)
N2—C33	1.457 (3)	C37—H37	0.9300
N2—C24	1.478 (3)	C38—C39	1.346 (7)
C1—C2	1.377 (4)	C38—H38	0.9300
C1—C6	1.379 (4)	C39—C40	1.387 (5)
C2—C3	1.386 (4)	C39—H39	0.9300
C2—H2	0.9300	C40—H40	0.9300
C3—C4	1.362 (5)	C41—C43	1.520 (3)
C3—H3	0.9300	C41—C42	1.566 (3)
C4—C5	1.373 (5)	C41—H41	0.9800
C4—H4	0.9300	C42—C44	1.501 (3)
C5—C6	1.386 (4)	C42—H42	0.9800
C5—H5	0.9300	C44—C45	1.383 (4)
C6—H6	0.9300	C44—C49	1.385 (4)
C7—C9	1.540 (3)	C45—C46	1.397 (5)
C7—C8	1.565 (3)	C45—H45	0.9300
C7—H7	0.9800	C46—C47	1.355 (6)
C8—C10	1.498 (3)	C46—H46	0.9300
C8—H8	0.9800	C47—C48	1.375 (6)
C10—C11	1.375 (3)	C47—H47	0.9300
C10—C15	1.396 (3)	C48—C49	1.392 (4)
C11—C12	1.382 (5)	C48—H48	0.9300
C11—H11	0.9300	C50—C51	1.493 (5)
C12—C13	1.355 (5)	C50—H50A	0.9700
C12—H12	0.9300	C50—H50B	0.9700
C13—C14	1.389 (5)	C51—H51A	0.9700
C13—H13	0.9300	C51—H51B	0.9700
C14—C15	1.390 (4)	C52—C53	1.389 (4)
C14—H14	0.9300	C52—C57	1.389 (3)
C16—C17	1.487 (4)	C53—C54	1.363 (4)
C16—H16A	0.9700	C53—H53	0.9300
C16—H16B	0.9700	C54—C55	1.366 (4)
C17—H17A	0.9700	C54—H54	0.9300
C17—H17B	0.9700	C55—C56	1.387 (4)
C18—C19	1.387 (3)	C55—H55	0.9300
C18—C23	1.393 (3)	C56—C57	1.381 (3)
C19—C20	1.368 (4)	C56—H56	0.9300
C19—H19	0.9300	C57—C58	1.505 (3)
C20—C21	1.373 (4)	C58—C59	1.565 (3)
C20—H20	0.9300	C58—H58	0.9800
C21—C22	1.393 (4)	C59—C60	1.538 (3)
C21—H21	0.9300	C59—H59	0.9800
C22—C23	1.383 (3)	C61—C66	1.369 (3)
C22—H22	0.9300	C61—C62	1.374 (3)
C23—C24	1.506 (3)	C62—C63	1.374 (3)
C24—C25	1.569 (3)	C62—H62	0.9300
C24—H24	0.9800	C63—C64	1.366 (4)
C25—C26	1.544 (3)	C63—H63	0.9300
C25—H25	0.9800	C64—C65	1.367 (3)
C27—C32	1.372 (3)	C64—H64	0.9300
C27—C28	1.382 (3)	C65—C66	1.391 (3)
C28—C29	1.380 (3)	C65—H65	0.9300
C28—H28	0.9300	C66—H66	0.9300
C29—C30	1.374 (4)	C67—C68	1.522 (3)
C29—H29	0.9300	C67—H67A	0.9700
C30—C31	1.366 (3)	C67—H67B	0.9700
C30—H30	0.9300	C68—H68A	0.9700
C31—C32	1.382 (3)	C68—H68B	0.9700
C31—H31	0.9300	O13—C70	1.319 (4)
C32—H32	0.9300	O13—C71	1.464 (4)
C33—C34	1.524 (3)	O14—C70	1.216 (4)
C33—H33A	0.9700	C69—C70	1.484 (5)
C33—H33B	0.9700	C69—H69A	0.9600
C34—H34A	0.9700	C69—H69B	0.9600
C34—H34B	0.9700	C69—H69C	0.9600
O7—C60	1.216 (3)	C71—C72	1.428 (6)
O8—C43	1.212 (3)	C71—H71A	0.9700
O9—C52	1.367 (3)	C71—H71B	0.9700
O9—C51	1.429 (3)	C72—H72A	0.9600
O10—C49	1.369 (4)	C72—H72B	0.9600
O10—C50	1.434 (4)	C72—H72C	0.9600
O11—C61	1.386 (2)		
			
C18—O3—C17	118.4 (2)	C40—C35—O12	124.3 (3)
C15—O4—C16	120.3 (2)	C36—C35—O12	114.4 (3)
C27—O5—C25	116.36 (16)	C35—C36—C37	117.3 (4)
C1—O6—C7	116.40 (18)	C35—C36—H36	121.3
C9—N1—C34	130.60 (19)	C37—C36—H36	121.3
C9—N1—C8	96.39 (16)	C38—C37—C36	122.3 (5)
C34—N1—C8	132.96 (17)	C38—C37—H37	118.8
C26—N2—C33	129.37 (19)	C36—C37—H37	118.8
C26—N2—C24	96.51 (16)	C39—C38—C37	118.0 (5)
C33—N2—C24	133.49 (17)	C39—C38—H38	121.0
C2—C1—C6	121.0 (2)	C37—C38—H38	121.0
C2—C1—O6	122.6 (2)	C38—C39—C40	122.2 (5)
C6—C1—O6	116.4 (3)	C38—C39—H39	118.9
C1—C2—C3	119.5 (3)	C40—C39—H39	118.9
C1—C2—H2	120.3	C35—C40—C39	118.7 (4)
C3—C2—H2	120.3	C35—C40—H40	120.6
C4—C3—C2	120.3 (4)	C39—C40—H40	120.6
C4—C3—H3	119.9	O12—C41—C43	116.36 (19)
C2—C3—H3	119.9	O12—C41—C42	118.80 (19)
C3—C4—C5	119.7 (3)	C43—C41—C42	85.36 (16)
C3—C4—H4	120.1	O12—C41—H41	111.3
C5—C4—H4	120.1	C43—C41—H41	111.3
C4—C5—C6	121.4 (4)	C42—C41—H41	111.3
C4—C5—H5	119.3	N3—C42—C44	115.9 (2)
C6—C5—H5	119.3	N3—C42—C41	85.74 (16)
C1—C6—C5	118.1 (3)	C44—C42—C41	115.83 (18)
C1—C6—H6	121.0	N3—C42—H42	112.3
C5—C6—H6	121.0	C44—C42—H42	112.3
O6—C7—C9	119.80 (17)	C41—C42—H42	112.3
O6—C7—C8	113.81 (18)	O8—C43—N3	132.2 (2)
C9—C7—C8	85.39 (15)	O8—C43—C41	136.1 (2)
O6—C7—H7	111.8	N3—C43—C41	91.69 (17)
C9—C7—H7	111.8	C45—C44—C49	118.2 (3)
C8—C7—H7	111.8	C45—C44—C42	122.8 (3)
N1—C8—C10	115.24 (18)	C49—C44—C42	119.0 (2)
N1—C8—C7	86.23 (15)	C44—C45—C46	120.8 (3)
C10—C8—C7	116.93 (17)	C44—C45—H45	119.6
N1—C8—H8	112.0	C46—C45—H45	119.6
C10—C8—H8	112.0	C47—C46—C45	119.9 (4)
C7—C8—H8	112.0	C47—C46—H46	120.0
O2—C9—N1	131.5 (2)	C45—C46—H46	120.0
O2—C9—C7	137.0 (2)	C46—C47—C48	120.6 (4)
N1—C9—C7	91.51 (17)	C46—C47—H47	119.7
C11—C10—C15	118.2 (3)	C48—C47—H47	119.7
C11—C10—C8	123.2 (2)	C47—C48—C49	119.5 (4)
C15—C10—C8	118.6 (2)	C47—C48—H48	120.2
C10—C11—C12	121.1 (3)	C49—C48—H48	120.2
C10—C11—H11	119.5	O10—C49—C44	115.1 (2)
C12—C11—H11	119.5	O10—C49—C48	123.9 (3)
C13—C12—C11	120.2 (3)	C44—C49—C48	120.8 (3)
C13—C12—H12	119.9	O10—C50—C51	106.7 (3)
C11—C12—H12	119.9	O10—C50—H50A	110.4
C12—C13—C14	121.1 (3)	C51—C50—H50A	110.4
C12—C13—H13	119.5	O10—C50—H50B	110.4
C14—C13—H13	119.5	C51—C50—H50B	110.4
C13—C14—C15	118.3 (3)	H50A—C50—H50B	108.6
C13—C14—H14	120.9	O9—C51—C50	106.1 (3)
C15—C14—H14	120.9	O9—C51—H51A	110.5
O4—C15—C14	123.7 (3)	C50—C51—H51A	110.5
O4—C15—C10	114.9 (2)	O9—C51—H51B	110.5
C14—C15—C10	121.2 (3)	C50—C51—H51B	110.5
O4—C16—C17	106.5 (2)	H51A—C51—H51B	108.7
O4—C16—H16A	110.4	O9—C52—C53	123.6 (2)
C17—C16—H16A	110.4	O9—C52—C57	116.1 (2)
O4—C16—H16B	110.4	C53—C52—C57	120.4 (3)
C17—C16—H16B	110.4	C54—C53—C52	120.1 (3)
H16A—C16—H16B	108.6	C54—C53—H53	120.0
O3—C17—C16	106.4 (2)	C52—C53—H53	120.0
O3—C17—H17A	110.4	C53—C54—C55	120.7 (3)
C16—C17—H17A	110.4	C53—C54—H54	119.6
O3—C17—H17B	110.4	C55—C54—H54	119.6
C16—C17—H17B	110.4	C54—C55—C56	119.4 (3)
H17A—C17—H17B	108.6	C54—C55—H55	120.3
O3—C18—C19	122.8 (2)	C56—C55—H55	120.3
O3—C18—C23	116.1 (2)	C57—C56—C55	121.3 (3)
C19—C18—C23	121.0 (2)	C57—C56—H56	119.3
C20—C19—C18	119.5 (3)	C55—C56—H56	119.4
C20—C19—H19	120.2	C56—C57—C52	118.2 (2)
C18—C19—H19	120.2	C56—C57—C58	121.3 (2)
C19—C20—C21	121.1 (3)	C52—C57—C58	120.6 (2)
C19—C20—H20	119.5	N4—C58—C57	114.02 (18)
C21—C20—H20	119.5	N4—C58—C59	85.72 (15)
C20—C21—C22	119.1 (3)	C57—C58—C59	115.33 (17)
C20—C21—H21	120.4	N4—C58—H58	113.0
C22—C21—H21	120.4	C57—C58—H58	113.0
C23—C22—C21	121.2 (2)	C59—C58—H58	113.0
C23—C22—H22	119.4	O11—C59—C60	120.8 (2)
C21—C22—H22	119.4	O11—C59—C58	113.64 (17)
C22—C23—C18	118.0 (2)	C60—C59—C58	85.43 (15)
C22—C23—C24	121.6 (2)	O11—C59—H59	111.5
C18—C23—C24	120.4 (2)	C60—C59—H59	111.5
N2—C24—C23	114.57 (18)	C58—C59—H59	111.5
N2—C24—C25	85.82 (15)	O7—C60—N4	132.2 (2)
C23—C24—C25	116.63 (17)	O7—C60—C59	136.5 (2)
N2—C24—H24	112.4	N4—C60—C59	91.30 (18)
C23—C24—H24	112.4	C66—C61—C62	119.9 (2)
C25—C24—H24	112.4	C66—C61—O11	123.28 (18)
O5—C25—C26	119.82 (19)	C62—C61—O11	116.8 (2)
O5—C25—C24	113.38 (17)	C61—C62—C63	119.7 (2)
C26—C25—C24	85.41 (15)	C61—C62—H62	120.2
O5—C25—H25	111.9	C63—C62—H62	120.2
C26—C25—H25	111.9	C64—C63—C62	121.1 (2)
C24—C25—H25	111.9	C64—C63—H63	119.4
O1—C26—N2	132.0 (2)	C62—C63—H63	119.4
O1—C26—C25	136.64 (19)	C63—C64—C65	119.3 (2)
N2—C26—C25	91.35 (17)	C63—C64—H64	120.3
C32—C27—C28	119.9 (2)	C65—C64—H64	120.3
C32—C27—O5	123.30 (19)	C64—C65—C66	120.2 (2)
C28—C27—O5	116.8 (2)	C64—C65—H65	119.9
C29—C28—C27	119.5 (2)	C66—C65—H65	119.9
C29—C28—H28	120.3	C61—C66—C65	119.8 (2)
C27—C28—H28	120.3	C61—C66—H66	120.1
C30—C29—C28	120.9 (2)	C65—C66—H66	120.1
C30—C29—H29	119.6	N4—C67—C68	115.0 (2)
C28—C29—H29	119.6	N4—C67—H67A	108.5
C31—C30—C29	119.1 (2)	C68—C67—H67A	108.5
C31—C30—H30	120.4	N4—C67—H67B	108.5
C29—C30—H30	120.4	C68—C67—H67B	108.5
C30—C31—C32	120.8 (2)	H67A—C67—H67B	107.5
C30—C31—H31	119.6	N3—C68—C67	115.5 (2)
C32—C31—H31	119.6	N3—C68—H68A	108.4
C27—C32—C31	119.8 (2)	C67—C68—H68A	108.4
C27—C32—H32	120.1	N3—C68—H68B	108.4
C31—C32—H32	120.1	C67—C68—H68B	108.4
N2—C33—C34	114.7 (2)	H68A—C68—H68B	107.5
N2—C33—H33A	108.6	C70—O13—C71	115.7 (3)
C34—C33—H33A	108.6	C70—C69—H69A	109.5
N2—C33—H33B	108.6	C70—C69—H69B	109.5
C34—C33—H33B	108.6	H69A—C69—H69B	109.5
H33A—C33—H33B	107.6	C70—C69—H69C	109.5
N1—C34—C33	114.73 (18)	H69A—C69—H69C	109.5
N1—C34—H34A	108.6	H69B—C69—H69C	109.5
C33—C34—H34A	108.6	O14—C70—O13	121.4 (4)
N1—C34—H34B	108.6	O14—C70—C69	126.2 (4)
C33—C34—H34B	108.6	O13—C70—C69	112.3 (3)
H34A—C34—H34B	107.6	C72—C71—O13	106.2 (4)
C52—O9—C51	118.7 (2)	C72—C71—H71A	110.5
C49—O10—C50	118.9 (2)	O13—C71—H71A	110.5
C61—O11—C59	116.70 (16)	C72—C71—H71B	110.5
C35—O12—C41	117.5 (2)	O13—C71—H71B	110.5
C43—N3—C68	130.9 (2)	H71A—C71—H71B	108.7
C43—N3—C42	96.04 (17)	C71—C72—H72A	109.5
C68—N3—C42	133.09 (18)	C71—C72—H72B	109.5
C60—N4—C67	131.1 (2)	H72A—C72—H72B	109.5
C60—N4—C58	96.23 (16)	C71—C72—H72C	109.5
C67—N4—C58	132.03 (17)	H72A—C72—H72C	109.5
C40—C35—C36	121.3 (3)	H72B—C72—H72C	109.5
			
C7—O6—C1—C2	39.0 (3)	C40—C35—C36—C37	−0.4 (5)
C7—O6—C1—C6	−143.2 (2)	O12—C35—C36—C37	179.8 (3)
C6—C1—C2—C3	0.5 (4)	C35—C36—C37—C38	−1.9 (7)
O6—C1—C2—C3	178.2 (2)	C36—C37—C38—C39	2.6 (9)
C1—C2—C3—C4	0.4 (5)	C37—C38—C39—C40	−1.0 (8)
C2—C3—C4—C5	−1.4 (5)	C36—C35—C40—C39	1.9 (5)
C3—C4—C5—C6	1.5 (6)	O12—C35—C40—C39	−178.4 (3)
C2—C1—C6—C5	−0.4 (4)	C38—C39—C40—C35	−1.2 (7)
O6—C1—C6—C5	−178.2 (3)	C35—O12—C41—C43	150.9 (2)
C4—C5—C6—C1	−0.6 (5)	C35—O12—C41—C42	−109.3 (2)
C1—O6—C7—C9	−88.7 (2)	C43—N3—C42—C44	−108.3 (2)
C1—O6—C7—C8	172.60 (19)	C68—N3—C42—C44	71.3 (3)
C9—N1—C8—C10	112.63 (19)	C43—N3—C42—C41	8.43 (18)
C34—N1—C8—C10	−69.8 (3)	C68—N3—C42—C41	−172.0 (3)
C9—N1—C8—C7	−5.46 (17)	O12—C41—C42—N3	−125.1 (2)
C34—N1—C8—C7	172.1 (2)	C43—C41—C42—N3	−7.46 (16)
O6—C7—C8—N1	125.40 (18)	O12—C41—C42—C44	−8.3 (3)
C9—C7—C8—N1	4.78 (15)	C43—C41—C42—C44	109.4 (2)
O6—C7—C8—C10	8.9 (3)	C68—N3—C43—O8	−6.5 (5)
C9—C7—C8—C10	−111.7 (2)	C42—N3—C43—O8	173.1 (3)
C34—N1—C9—O2	7.3 (4)	C68—N3—C43—C41	171.8 (2)
C8—N1—C9—O2	−175.1 (3)	C42—N3—C43—C41	−8.66 (18)
C34—N1—C9—C7	−172.1 (2)	O12—C41—C43—O8	−53.8 (4)
C8—N1—C9—C7	5.54 (18)	C42—C41—C43—O8	−173.8 (3)
O6—C7—C9—O2	60.6 (4)	O12—C41—C43—N3	128.0 (2)
C8—C7—C9—O2	175.5 (3)	C42—C41—C43—N3	8.07 (17)
O6—C7—C9—N1	−120.1 (2)	N3—C42—C44—C45	15.0 (3)
C8—C7—C9—N1	−5.19 (16)	C41—C42—C44—C45	−83.2 (3)
N1—C8—C10—C11	−18.3 (3)	N3—C42—C44—C49	−168.2 (2)
C7—C8—C10—C11	80.8 (3)	C41—C42—C44—C49	93.5 (3)
N1—C8—C10—C15	163.82 (19)	C49—C44—C45—C46	0.9 (4)
C7—C8—C10—C15	−97.1 (2)	C42—C44—C45—C46	177.6 (2)
C15—C10—C11—C12	−0.7 (4)	C44—C45—C46—C47	2.0 (4)
C8—C10—C11—C12	−178.5 (3)	C45—C46—C47—C48	−3.3 (5)
C10—C11—C12—C13	−1.0 (5)	C46—C47—C48—C49	1.7 (5)
C11—C12—C13—C14	1.1 (6)	C50—O10—C49—C44	142.0 (3)
C12—C13—C14—C15	0.5 (5)	C50—O10—C49—C48	−42.6 (4)
C16—O4—C15—C14	32.2 (4)	C45—C44—C49—O10	173.0 (2)
C16—O4—C15—C10	−151.8 (2)	C42—C44—C49—O10	−3.9 (3)
C13—C14—C15—O4	173.6 (3)	C45—C44—C49—C48	−2.5 (4)
C13—C14—C15—C10	−2.2 (4)	C42—C44—C49—C48	−179.4 (2)
C11—C10—C15—O4	−173.8 (2)	C47—C48—C49—O10	−173.8 (3)
C8—C10—C15—O4	4.1 (3)	C47—C48—C49—C44	1.3 (5)
C11—C10—C15—C14	2.3 (4)	C49—O10—C50—C51	−130.4 (3)
C8—C10—C15—C14	−179.8 (2)	C52—O9—C51—C50	−164.1 (2)
C15—O4—C16—C17	134.4 (3)	O10—C50—C51—O9	66.6 (3)
C18—O3—C17—C16	153.4 (2)	C51—O9—C52—C53	−15.0 (4)
O4—C16—C17—O3	−68.4 (3)	C51—O9—C52—C57	164.8 (2)
C17—O3—C18—C19	27.5 (3)	O9—C52—C53—C54	179.3 (2)
C17—O3—C18—C23	−154.7 (2)	C57—C52—C53—C54	−0.5 (4)
O3—C18—C19—C20	177.7 (2)	C52—C53—C54—C55	0.4 (4)
C23—C18—C19—C20	0.0 (3)	C53—C54—C55—C56	−0.4 (4)
C18—C19—C20—C21	−0.2 (3)	C54—C55—C56—C57	0.6 (4)
C19—C20—C21—C22	0.2 (4)	C55—C56—C57—C52	−0.7 (3)
C20—C21—C22—C23	0.1 (3)	C55—C56—C57—C58	177.1 (2)
C21—C22—C23—C18	−0.2 (3)	O9—C52—C57—C56	−179.2 (2)
C21—C22—C23—C24	179.76 (19)	C53—C52—C57—C56	0.6 (3)
O3—C18—C23—C22	−177.61 (18)	O9—C52—C57—C58	3.0 (3)
C19—C18—C23—C22	0.2 (3)	C53—C52—C57—C58	−177.2 (2)
O3—C18—C23—C24	2.4 (3)	C60—N4—C58—C57	−106.8 (2)
C19—C18—C23—C24	−179.81 (18)	C67—N4—C58—C57	64.9 (3)
C26—N2—C24—C23	109.9 (2)	C60—N4—C58—C59	9.08 (18)
C33—N2—C24—C23	−61.3 (3)	C67—N4—C58—C59	−179.2 (2)
C26—N2—C24—C25	−7.52 (18)	C56—C57—C58—N4	36.9 (3)
C33—N2—C24—C25	−178.7 (2)	C52—C57—C58—N4	−145.29 (19)
C22—C23—C24—N2	−32.3 (3)	C56—C57—C58—C59	−60.0 (3)
C18—C23—C24—N2	147.69 (18)	C52—C57—C58—C59	117.8 (2)
C22—C23—C24—C25	65.7 (3)	C61—O11—C59—C60	89.5 (2)
C18—C23—C24—C25	−114.3 (2)	C61—O11—C59—C58	−171.27 (19)
C27—O5—C25—C26	−85.4 (2)	N4—C58—C59—O11	−129.7 (2)
C27—O5—C25—C24	176.11 (19)	C57—C58—C59—O11	−15.1 (3)
N2—C24—C25—O5	127.13 (19)	N4—C58—C59—C60	−7.93 (16)
C23—C24—C25—O5	11.7 (3)	C57—C58—C59—C60	106.7 (2)
N2—C24—C25—C26	6.54 (16)	C67—N4—C60—O7	−1.0 (5)
C23—C24—C25—C26	−108.9 (2)	C58—N4—C60—O7	170.9 (3)
C33—N2—C26—O1	−1.5 (4)	C67—N4—C60—C59	178.9 (2)
C24—N2—C26—O1	−173.3 (3)	C58—N4—C60—C59	−9.21 (18)
C33—N2—C26—C25	179.4 (2)	O11—C59—C60—O7	−56.5 (4)
C24—N2—C26—C25	7.62 (18)	C58—C59—C60—O7	−171.4 (3)
O5—C25—C26—O1	59.4 (4)	O11—C59—C60—N4	123.5 (2)
C24—C25—C26—O1	173.8 (3)	C58—C59—C60—N4	8.66 (17)
O5—C25—C26—N2	−121.5 (2)	C59—O11—C61—C66	−40.3 (3)
C24—C25—C26—N2	−7.15 (17)	C59—O11—C61—C62	142.4 (2)
C25—O5—C27—C32	42.8 (3)	C66—C61—C62—C63	0.1 (4)
C25—O5—C27—C28	−139.8 (2)	O11—C61—C62—C63	177.4 (3)
C32—C27—C28—C29	−0.5 (4)	C61—C62—C63—C64	−0.1 (5)
O5—C27—C28—C29	−178.0 (3)	C62—C63—C64—C65	0.0 (5)
C27—C28—C29—C30	−0.2 (5)	C63—C64—C65—C66	0.2 (4)
C28—C29—C30—C31	0.8 (4)	C62—C61—C66—C65	0.1 (4)
C29—C30—C31—C32	−0.7 (4)	O11—C61—C66—C65	−177.0 (2)
C28—C27—C32—C31	0.6 (4)	C64—C65—C66—C61	−0.3 (4)
O5—C27—C32—C31	177.9 (2)	C60—N4—C67—C68	−112.9 (3)
C30—C31—C32—C27	0.0 (4)	C58—N4—C67—C68	78.0 (3)
C26—N2—C33—C34	107.1 (3)	C43—N3—C68—C67	−112.1 (3)
C24—N2—C33—C34	−84.2 (3)	C42—N3—C68—C67	68.5 (4)
C9—N1—C34—C33	112.5 (3)	N4—C67—C68—N3	−97.4 (3)
C8—N1—C34—C33	−64.3 (3)	C71—O13—C70—O14	2.0 (5)
N2—C33—C34—N1	95.2 (2)	C71—O13—C70—C69	−174.3 (4)
C41—O12—C35—C40	17.9 (4)	C70—O13—C71—C72	175.5 (4)
C41—O12—C35—C36	−162.4 (2)		

### Hydrogen-bond geometry (Å, °)

**Table:**

*D*—H···*A*	*D*—H	H···*A*	*D*···*A*	*D*—H···*A*
C53—H53···O6	0.93	2.52	3.344 (3)	147
C2—H2···O2	0.93	2.42	3.250 (3)	149
C32—H32···O1	0.93	2.33	3.178 (3)	151
C66—H66···O7	0.93	2.48	3.299 (3)	147
